# Supplementary material for: Chromothripsis during telomere crisis is independent of NHEJ, and consistent with a replicative origin
Source: Genome Res. 2019 May;29(5):737–49. doi: 10.1101/gr.240705.118 (PMC6499312; doi:10.1101/gr.240705.118)
Supplement: Supplemental Material [file supp_gr.240705.118_Supplemental_file_1.zip › contigs/annotated_contigs/DB48/contig.2.DB48_length_527_mean_cov_4.37191650854.docx]

**DB48_length_527_mean_cov_4.37191650854**

```
ACAATTTCTTTCACAAGCAGCCTACATTCAGTGTCTGATCAACACGTGAGATCAAGAAACAGATGCTTCACCTACTCCAGACCACTCAC
 >chr7:52533429-52533726 + E=4e-164
CTGTGGCTCACTCAGCACTCCCCATGGGTGTGTCCTGGGTCATTTTCCACTGCACCACAATGCACTATCTCCCCATGCCAAAGCCTCCT

GTCTCTCACAAGGCAGTGATCCCAATCATGCTTCCTCATAAGCATACTGCACACTAATTTTAATCTCAGATTTTGCTACCTGAAGGACC

TAACCCATGACACTTGGTGCCAAGAACGGT|GA|CTAGGAAATCGTGACTAGAAAACATTTAGGAATACCATTCTAAGTTAACTTTCAG
                                 >chr7:52531973-52532138 - E=1e-78
ACTTGCTTCAAATGTGAATTGAACCATCAGCAACTGAAGTTATAGAACAGTTTGAACTGAAAATAAAGTCAATTAATTGTAAAAAAAAA

AATGTAAAATTCGATCTTG|AATTCAAGGAAGAACGAATATAATGTCTTACAAACAAAATACCTGCATACTAAGTGTGCACAGTG
```
